# A Novel Computer-Aided Design/Computer-Aided Manufacturing (CAD/CAM) 3D Printing Method for Nasal Framework Reconstruction Using Microvascular Free Flaps

**DOI:** 10.7759/cureus.28971

**Published:** 2022-09-09

**Authors:** Carlos M Chiesa-Estomba, Jose González-García, Jon A Sistiaga-Suarez, Iago González Fernández

**Affiliations:** 1 Otolaryngology-Head and Neck Surgery, Hospital Universitario Donostia, San Sebastián, ESP; 2 Enginnering, Kune Implants, Vigo, ESP

**Keywords:** oncological reconstruction, three-dimensional (3d) printing, radial forearm free flap, plastic and reconstructive surgery, nose surgery

## Abstract

The need for a total or subtotal nose resection represents a dramatic situation for any patient and a challenge for the reconstructive surgeon. Because even in the most skillful hands, an optimal result may be difficult to achieve. In this way, sometimes a free flap reconstruction is needed. A subtotal nasal reconstruction is presented using a fasciocutaneous free flap supported by computer-aided design (CAD), computer-aided manufacturing (CAM), and novel 3D printed guide to recreate the nasal cartilage framework. A successfully free flap reconstruction of the nasal framework was achieved supported by the CAD/CAM 3D printed template. We present a novel approach for nasal total or subtotal reconstruction using a CAD/CAM 3D printed model to create the nasal framework, based on the anatomical and radiological evaluation of the patient.

## Introduction

The need for a total or subtotal nose resection is a dramatic situation for any patient. This is because even in the most skillful hands, optimal results may be difficult to achieve. Any surgical reconstruction aims to restore the functional and anatomical characteristics of the structure operated on. When we deal with nose reconstruction, there are three main elements that we need to consider to achieve a successful result, namely, the nasal lining, the structural support, and the external skin cover. On the other hand, the main functional target is to restore the nasal breathing route.

Nasal defects can be repaired with a variety of autologous techniques (e.g., local flaps, with cartilage or bone grafts) or with prosthetic implants; however, in some cases, the type of nasal defect, the lack of local tissue, a local flap failure, previous radiation therapy and/or the need to cover a large nasal defect make it necessary to use a customized free flap [1]. In this scenario, a prelaminated fasciocutaneous free flap with costal cartilage represents the most reliable option, radial- and ulnar-free flaps being the most commonly used [2]. On the other hand, in recent years, there has been growing interest among medical and surgical sub-specialties in the use of 3D printing technology for surgical planning and the development of biocompatible scaffolds [3].

Here, we report our experience with a patient who underwent a subtotal nasal reconstruction using a fasciocutaneous free flap supported by a novel computer-aided design (CAD)/computer-aided manufacturing (CAM) 3D printed guide, to recreate the cartilage framework of the nose.

## Technical report

We describe the case of a 38-year-old male patient who had undergone composite subtotal left nasal resection, reconstructed with a forehead flap and rib cartilage due to invasive squamous cell carcinoma of the left nostril, and had experienced a nasal retraction in the reconstructed region. Therefore, a secondary reconstruction strategy to improve the esthetic and functional result was developed and described in detail.

**Figure 1:**
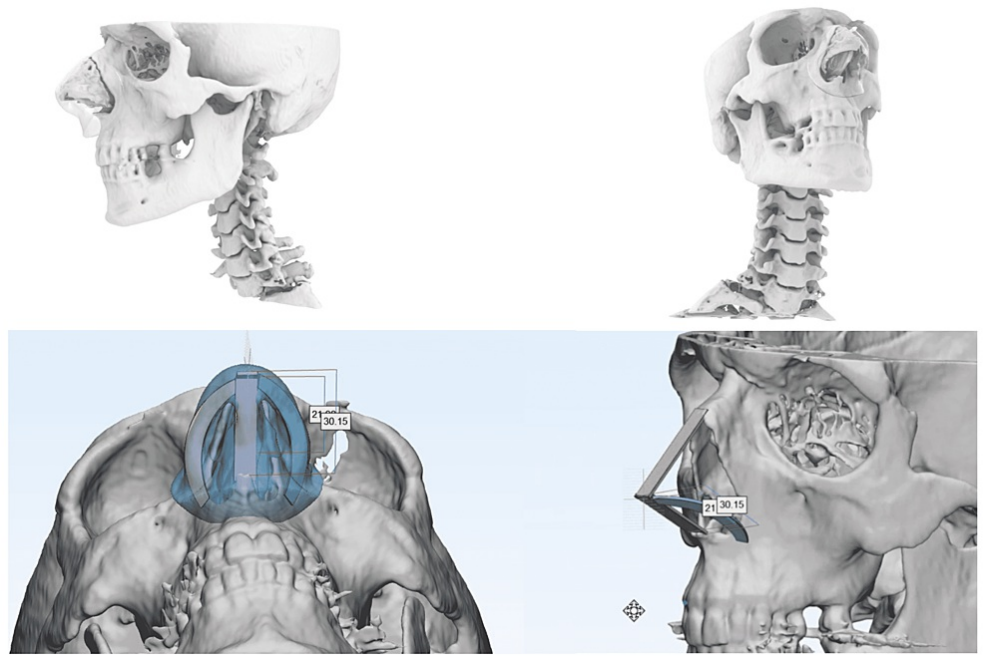
Three-dimensional anatomical reconstruction, nasal measurements, and designing of the shape and curvature of the rib grafts based on the patient's nasal anatomy.

To achieve our goal, we develop a three-staged reconstruction workflow and employed a novel method based on 3D printing technology. In the first stage, a CAD/CAM model was developed using preoperative nasal computed tomography scan (CT) data to improve rib cartilage contouring. The process begins with segmenting of medical image files (DICOM). The objective is to obtain a 3D anatomical reconstruction that is accurate to the patient’s preoperative condition. To perform the segmentation, it is necessary to use a segmentation method suitable for the patient's bone tissue, such as thresholding and algorithms for the segmentation of soft tissue. The planning CT images for this case reported are presented in Figure 1. Once the patient’s exact anatomy had been extracted from the CT scan, the resulting 3D solids were exported to the design software, in this case, Materialise 3-matic (3DZ Treviso: Castelfranco Veneto, Italy). The software allows us to obtain the exact measurements, shape, and curvature of the rib grafts to be designed based on the patient's nasal anatomy, with the goal of restoring not only function but also appearance. Figure 1 also shows the result of the design of the grafts superimposed on the patient's anatomy.

**Figure 2:**
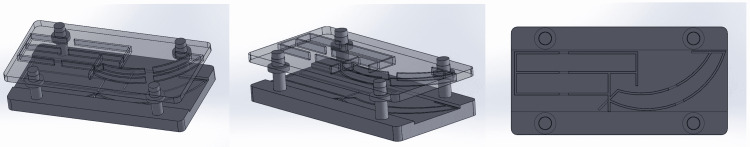
Three-dimensional (3D) model printed using a sterilizable polymer.

With all the anatomical information on the different rib cartilage grafts transferred to the CAD design program, an exact cutting guide is created to help the surgeon conduct the surgery accurately in accordance with the surgical plan. The width of the surgical guide should correspond to the maximum removable area of the costal cartilage, considering manufacturing tolerances, the surgeon's comfort, and the mechanical properties of the tissue to be cut. For this purpose, it was decided to use a removable guide arrangement with two plates connected with pillars which give the structure sufficient rigidity to allow the surgeon to operate on the costal cartilage. It was considered that the graft to be operated on should have two different thicknesses and it should be possible to bring the plates close enough to put pressure on the tissue and hold it in place. For this reason, two positions were created in the joining pillars, to allow the distance between the upper and lower plates to be set depending on the thickness of the cartilage to be cut. Specifically, the position of the upper plate is changed by rotating the support pillars by 180 degrees. Finally, the 3D model was printed using a sterilizable polymer, polyamide 12 (Figure 2).

**Figure 3:**
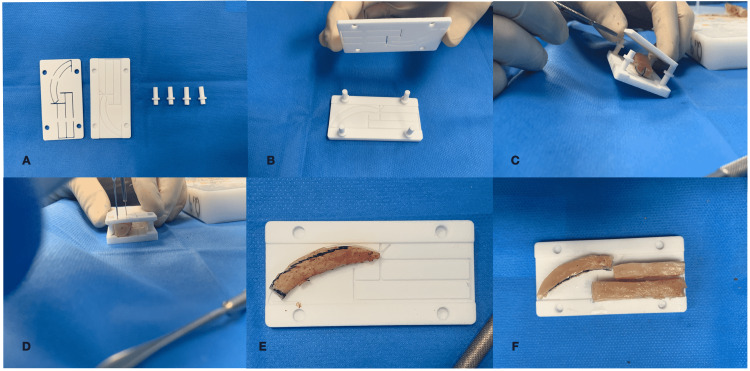
Cartilage support obtained using the sterilized rubber 3D CAD/CAM template. The images show (A) 3D model, (B) 3D model with connecting pillars, (C) costal rib inside the 3D model, (D) knife cartilage cutting, (E) alar-cartilage design, and (F) cartilage framework parts. CAD/CAM: computer-aided design/computer-aided manufacturing

In the second stage, a prelaminated radial forearm free flap with a second rib cartilage graft was performed. The radial forearm free flap was elevated distally following the classical technique. Seeking to improve the esthetic result, the refined cartilage shaping enabled shaping of the columella, and left alar and vertical cartilage support were developed based on a sterilized rubber 3D CAD/CAM mold, which served as a template. Then, the neo-cartilage structures were inserted into the free flap in the subcutaneous plane. The fasciocutaneous defect in the arm was skin grafted, and the coverage was removed after 14 days (Figure 3).

**Figure 4:**
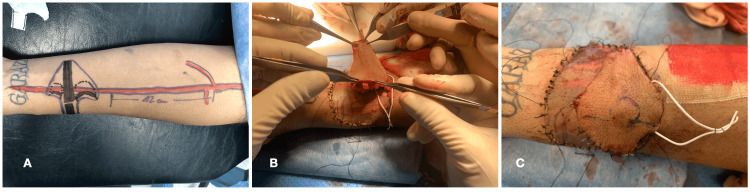
Surgical design in the left arm of the patient (A), neo-cartilage structures insertion in the free flap in the subcutaneous plane (B), and the fasciocutaneous defect in the arm was skin grafted and the neo-nose covered (C).

In the third stage, one month later, the neo-nose viability was confirmed, as was an excellent vascular supply in the arm of the patient (Figures 4A-4C). At this stage, the creation of the radial forearm free flap (neo-nose) partially raised previously was completed, with a 14-cm vascular pedicle. Simultaneously, the left facial artery and vein were dissected, and after tunneling the pedicle under the cheek tissue, an end-to-end anastomosis was performed using interrupted 8-0 sutures in both the artery and vein. For cartilage framework fixation, titanium microplates and screws were used and fixed to the nasal spine and remanent nasal bone.

**Figure 5:**
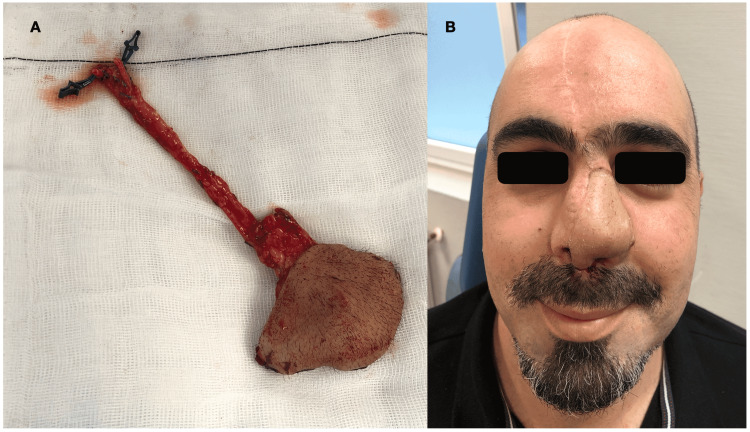
Radial forearm free flap (neo-nose) with 14 cm of vascular pedicle (A) and final result six weeks after surgery (B).

A nasal splint was used for one month to maintain long-term patency of both nostrils, with an acceptable final esthetic result, an adequate symmetry of the nostrils, and correct tip projection (Figures 5A, 5B). Moreover, the patient was satisfied with the esthetic result.

## Discussion

The causes of nasal defects can be categorized as follows: traumatic, post-ablative (due to malignancies), infection, cocaine abuse, Wegener granulomatosis, and congenital arrhinia [4]. In oncologic resections, a combination of procedures is usually necessary to completely repair subtotal or total nasal defects. Contouring and secondary revisions may be required to enhance the post-operative functional and esthetic outcomes of reconstruction.

Multiple approaches have been described to restore nasal appearance after total or subtotal reconstruction. Regardless of the etiology, nasal defects can be restored with a variety of techniques including local flaps, free flaps, with or without cartilage or bone grafts, or composite grafts, as well as prosthetic implants.

Regarding radial forearm flaps, three major variations have been described for nasal reconstruction - infolding [5], multiple skin paddles [6], or prelaminated [7]. In our case, we used the prelaminated approach to recreate the nose structure, because we were able to include the cartilage framework in the subcutaneous plane and evaluate integration and vascularization before transferring the flap to the face.

On the other hand, one of the main factors influencing the final result in patients undergoing nasal reconstruction is the framework. Usually, a supporting nasal framework is created using non-vascularized grafts (bone or cartilage), without a blood supply, and with this approach, survival depends on surrounding tissue vascularization [8]. Nevertheless, it is well-known that vascularized cartilage or bone transfer has significant advantages, due to preserved cellular viability, and the presence of periosteum and perichondrium [9].

In this report, we present a method that may help surgeons to obtain a reliable nose mold, as well as create a custom-made nasal framework. Recently, Walton et al. described the use of a 3D scaffold to serve as a support and form modulator for nasal reconstruction [3], based on porous polyethylene, highlighting its advantages including excellent biocompatibility, tissue ingrowth, and adherence [10,11]. Additionally, a previous approach using a CAD/CAM method was described by Ahcan et al. who used a prefabricated model to define the shape and contour of the flap [12]. In our case, we have incorporated a novel method for improving cartilage shaping using a CAD/CAM 3D printed model as a template for cartilage modeling.

## Conclusions

With the development of new technologies, prefabrication of a nasal framework using 3D printing technology based on preoperative patient anatomy is possible. In this manuscript, we present a novel approach for total or subtotal nasal reconstruction using a CAD/CAM 3D printed model to improve the shape of the nasal framework, based on an anatomical and radiological study of the patient. Though we performed a free flap reconstruction in this case, the approach proposed could also be used for local flaps.
